# A valid name for the Xishuangbanna gourd, a cucumber with carotene-rich fruits

**DOI:** 10.3897/phytokeys.85.17371

**Published:** 2017-08-31

**Authors:** Susanne S. Renner

**Affiliations:** 1 Systematic Botany and Mycology, Menzinger Str. 67, 80638 Munich, Germany

**Keywords:** China, Yunnan, cucumber, *Cucumis sativus*, plant breeding, genomics, valid name

## Abstract

Herbarium specimens deposited in publicly accessible collections are the basis for all scientific names because only permanent specimens can be re-studied by independent researchers, the very essence of science. Re-investigations may be done with morphological, chemical, genomic, computer-tomographic, or other methods. Based on new herbarium material, I here provide a name for the Xishuangbanna gourd, a plant long cultivated in Yunnan because of its large non-bitter fruits, rich in β-carotene. Genome re-sequencing of numerous accessions has shown that this cucumber mutant is closer to Cucumis sativus var. sativus than is the wild bitter-fruited progenitor *C. sativus* var. hardwickii, and two dozen studies have further clarified the genetics of key traits, including pulp color, fruit shape, and flowering times. Morphological and molecular diagnoses of the new variety are provided and museum-quality specimens have been distributed to the World’s major herbaria.

## Introduction

**Figure 1.**
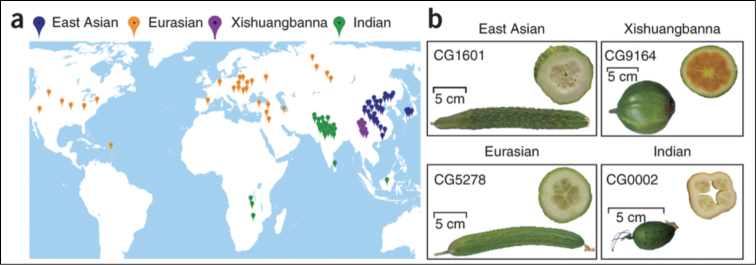
Cucumber populations. **a** The core collection of 115 lines re-sequenced by Qi et al. (2013). Colour codes indicate geographic groups **b** Fruit morphology of the four groups. The cucumber line CG1601 (East Asian) bears fruits with dense, white spines and an elongated stalk. Fruits of cucumber line CG5278 (Eurasian) lack spines and have a short fruit stalk. Cucumber line CG9164 (Xishuangbanna) bears melon-like fruits with a low fruit shape index (length/width) and a unique orange endocarp. Cucumber line CG0002 (Indian) bears small, oval fruits with sparse, black spines. Note that the images differ in scale. Reproduced from Qi et al. including Renner (2013).

**Figure 2.**
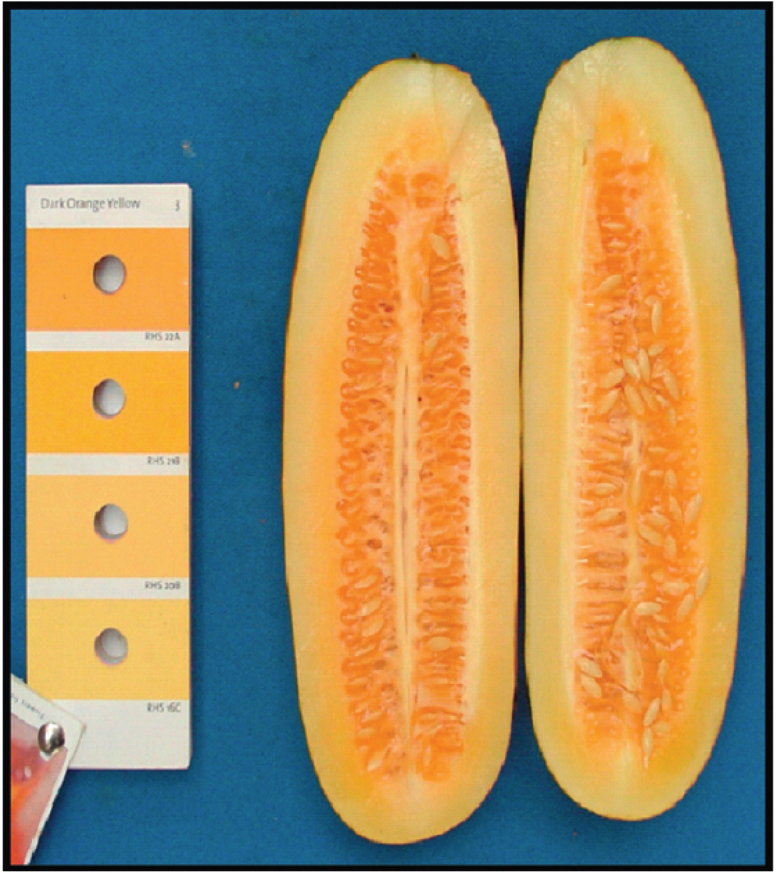
Section of a mature fruit of Cucumis sativus var. xishuangbannanensis. Reproduced from Staub et al. (2011).

Southern Yunnan has a rich flora, with elements of both Indian and Chinese tropical biota, and local farmers from different ethnic backgrounds have long exerted diversifying selection on plants domesticated in this region of Southeast Asia. One such crop is the cucumber, *Cucumis sativus* L., of which bitter-fruited progenitor populations (*C. sativus* var. hardwickii (Royle) Alef.) occur in the Himalayan foothills in India, Myanmar (Burma), North and West Thailand, and Southwest China (Royle 1839: plate 47 shows *C. hardwickii*; Naudin, 1859: p. 30 discusses *C. hardwickii*; Sebastian et al. 2010). Genome re-sequencing of 115 cucumber lines sampled from 3,342 accessions worldwide has revealed four deeply separated genetic clusters consisting of Eurasian, East Asian, Indian, and Xishuangbanna cucumbers (Qi et al. 2013; Fig. 1). The Xishuangbanna gourd or cucumber (both English names are used) has large cylindric or sub-globose smooth fruits and a pulp that at maturity resembles honey melon, *Cucumis melo* L., in color (Fig. 2). A single fruit can weigh 2–3 kg, and the seed number can exceed 1000 (Qi et al. 1983; Yang et al. 1991; Chen et al. 1994). No other variety of *C. sativus* has these traits.

The Xishuangbanna cucumber was brought to horticulturists’ attention by 1979–1980 investigations of crop cultivars of the Yunnan province (Yang et al. 1991), and Qi Chunzhang, Yuan Zhenzhen, and Li Yuxian in 1983 were the first to argue that this local form deserves a formal name so that insights about its traits can be shared widely. They provided three arguments for naming it as a variety of *C. sativus*: (i) It has *2n* = 14 chromosomes, just like *C. sativus*, but unlike the remaining species of *Cucumis* so far counted (Kirkbride 1993; Chen et al. 1999; Yang et al. 2012, 2013). (ii) Crosses between typical *C. sativus* and the Xishuangbanna plants are fertile. (iii) Peroxidase zymogramms of Xishuangbanna gourd and *C. melo* are quite different from each other.

Unfortunately, Qi and colleagues failed to make a type collection and to provide a Latin diagnosis or description, which in 1983 was still required for validly naming a plant taxon. No herbarium material is mentioned in their paper, but their text, geographic information, and B/W photos leave no doubt about which plant they are referring to. A search in the Chinese virtual herbarium (http://www.cvh.ac.cn/news/8) and correspondence with curators at KUN, IBSC, PE, and XTBG showed that no specimens have been deposited in these largest Chinese herbaria. The USDA’s National Plant Germplasm System (NPGS) has only germplasm PI 618931 of the Xishuangbanna gourd, but it is not available for distribution because it has proven difficult to regenerate (K. R. Reitsma, Curator of Vegetable Crops, North Central Regional Plant Introduction Station, Iowa State University, Ames, Iowa; personal communication on 9 June 2017). The Beijing Crop Germplasm Resources information system supported by the Vegetable Research Center (BVRC) maintains 1915 accessions labeled as ‘*Cucumis sativus*’ but provides no further taxonomic information; the database (icgr.caas.net.cn) supported by the Chinese Academy of Agricultural Sciences (CAAS) contains 1447 records for cucumber, again without further taxonomic data (Guo Shaogui, personal communication, 12 June 2017).

Based on newly prepared herbarium specimens from Xishuangbanna, I here provide a valid name for the Xishuangbanna cucumber and briefly discuss research results on its main traits.

## Methods

Plants were collected on fields in the Xishuangbanna region and nine duplicates dried between newspaper.

## Results

### Morphological diagnosis

#### Cucumis sativus L. var. xishuangbannanensis

Taxon classificationPlantaeCucurbitalesCucurbitaceae

Qi & Yuan ex S.S.Renner var. nov.

urn:lsid:ipni.org:names:77165361-1

##### Notes.

Differs from all other forms of *C. sativus* in producing thick-cylindric fruits that have ≥ 5 carpels and at maturity a non-bitter orange pulp (Fig. 2), while var. hardwickii and var. sativus both have 3 carpels and green pulp, which in var. hardwickii is bitter, in var. sativus non-bitter.

##### Molecular diagnosis.

**Figure 3.**
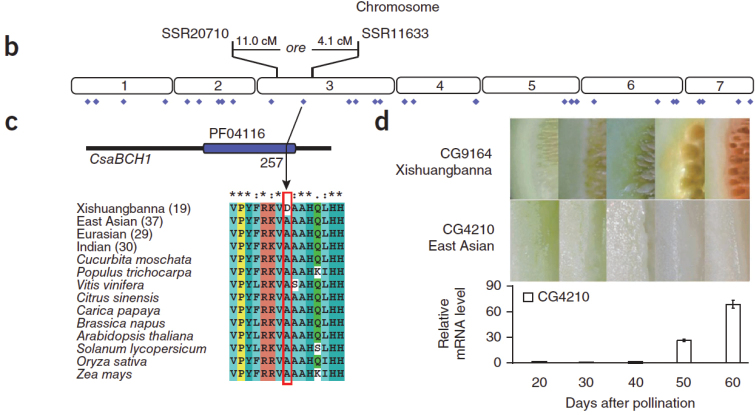
Physical position of the *ore* gene on *Cucumis sativus* chromosome 3. Blue diamonds below the seven chromosomes indicate the positions of 43 SNPs **c** Mutation at residue 257 changing the conserved amino acid of a putative β-carotene hydroxylase (*CsaBCH1*). Xishuangbanna group cucumbers carry asparagine, whereas all other cucumbers and homologous proteins from ten other species carry alanine **d** *CsaBCH1* mRNA levels in Xishuangbanna cucumbers that accumulate β-carotene. Reproduced from Qi et al. (2013).

Verifiable DNA differences (characters): On chromosome 3, within the physical interval that spans the *ore* gene, Xishuangbanna cucumbers carry asparagine, whereas all other *C. sativus* (37 from East Asia, 29 from Eurasia, 30 from India) and homologous proteins from ten other species of flowering plants carry alanine at this site. This amino acid change at residue 257 (p.Ala257Asp) in *Csa3G183920*, affects a gene encoding a putative β-carotene hydroxylase, designated *CsaBCH1* by Qi et al. (2013; Fig. 3).

##### Type.

CHINA, Yunnan Province, Xishuangbanna region, Menglun, Mengla county. Farmland of the Jinuo people at 1200 m, collected in flower on 18 July 2017; young fruits photographed on 20 July 2017 to show the pulp just beginning to turn orange; Chang Yanfen 1141 (holotype: PE; isotypes; IBSC, K, KUN, L, M, MO, US, XTBG).

##### Distribution.

China (Yunnan), Laos, Vietnam, probably also Myanmar/Burma.

Flowering in July, mature fruits from the end of August onward (personal observation by Chang Yanfen).

##### Habitat, cultivation, and use.

Growing in a tropical warm, humid climate above 1000 m alt. This form of cucumber has long been cultivated by the Jinuo, Hani, and Aini ethnic groups of China, Laos, and Vietnam, who call it ‘shihuo’ (Chen et al. 1994; Chang Yanfen, personal communication, July 2017) or ‘da huang gua’ (big cucumber) and ‘shan huang gua’ (mountain cucumber; Yang et al. 1991). Local farmers intercrop gourd plants with dry rice, and cultivate three regional types, called Cattle shihuo, Ivory shihuo, and Round shihuo (Chen et al. 1994). They sow the seeds in April and harvest fruits from August to October/November. The Xishuangbanna gourd has primary stems 6–7 meters long and 20–40 lateral branches; plants are monoecious, and nodes often bear one female and one male flower (Chen et al. 1994). A single plant can bear about 10 mature fruits with a yield of 10–20 kilograms per plant (Chen et al. 1994). Like other cucumbers in China, the fruits are eaten raw or boiled, sliced, and spiced (Yang et al. 1991; Chen et al. 1994).

##### Etymology.

The epithet was proposed by Qi et al. (1983) and refers to the geographic occurrence.

##### Specimens examined.

The monograph of *Cucumis* by Kirkbride (1993) mentions Qi et al.’s (1983) paper on the Xishuangbanna cucumber in the discussion following *C. sativus* (with the erroneous spelling ‘*xishuangbannanesis*’ of the original paper), but does not formally treat the name because Kirkbride, of course, knew that the name was not valid for lack of a type and a Latin diagnosis or description. KUN has three specimens from Yunnan of which Kirkbride in 1991 annotated one as ‘*C. sativus*’, while the other two are annotated by Chinese taxonomists as var. hardwickii. Without mature fruits (whose carpel number could be determined) or DNA sequencing, it cannot be decided whether any of these specimen might represent the orange cucumber. I have not found any herbarium specimens annotated as ‘var. xishuangbannanensis’ despite numerous emails (cf. Acknowledgements).

## Discussion

Cucumis sativus var. xishuangbannanensis has an orange endocarp high in carotenoids (Qi et al. 1983; Navazio 1994, Simon and Navazio 1997, Navazio and Simon 2001; Staub et al. 1999; McCreight et al. 2013). Efforts to incorporate genetic variation found in the Chinese material into U.S. cucumber germplasm to improve human health (Staub et al. 2011) have met with limited success. The orange fruit pulp is due to the accumulation of β-carotene (i.e., provitamin A), and the inheritance of this trait is by now well understood (Navazio 1994; Navazio and Simon 2001; Cuevas et al. 2010; Shen et al. 2011; Bo et al. 2012; Qi et al. 2013; Lu et al. 2015): Two recessive genes control the β-carotene content in the mesocarp, while one recessive gene controls β-carotene content in the endocarp (Cuevas et al. 2010). In the most extensive study so far, Qi et al. (2013) re-sequenced 115 *C. sativus* accessions from central and western Asia, Europe, the United States, and the Xishuangbanna region and found that a single SNP, resulting in an amino acid change at residue 257 (pAla257Asp) in *Csa3G183920*, modifies a gene encoding a putative β-carotene hydroxylase, designated *CsaBCH1* (Fig. 3), which is upregulated during the maturation of Xishuangbanna cucumbers so that 40–60 days after pollination, fruits rapidly accumulate β-carotene (Qi et al. 2013; Fig. 3).

Flowering time and fruit size variation in the Xishuangbanna cucumber have also been studied, and a QTL analysis implicated 11 QTLs on two chromosomes in determining photoperiod-dependent flowering time and the round fruit shape (Qu et al. 2014; Pan et al. 2017). The short hypocotyl is controlled by a recessive allele (Bo et al. 2016), and the carpel number of usually 5, not three carpels as in var. hardwickii and var. sativus, is controlled by a single gene for which a candidate locus has been identified (Li et al. 2016).

Concerning the time of domestication of the Xishuangbanna cucumber, synteny analyses among *C. sativus* var. sativus, var. hardwickii, and var. xishuangbannanensis have revealed that the Xishuangbanna cucumber shares major chromosomal rearrangements in chromosomes 4, 5, and 7 with var. sativus but not var. hardwickii, suggesting that it originated through diversifying selection after cucumber domestication (Bo et al. 2015). Comparison of fluorescence *in situ* hybridization (FISH) patterns in the three varieties also supports these relationships (Zhao et al. 2011: this study misspells the varietal name *xishuangbannanensis*). The sister species of *C. sativus* is *C. hystrix*, which has 12 (not seven like *C. sativus*), chromosomes (Chen et al. 1999), and both these species belong to an Asian/Australian clade of the genus *Cucumis* (Renner et al. 2007). A bottleneck that could have occurred during the initial domestication of the Xishuangbanna cucumber mutant has been dated to 3450 years ago (Qi et al. 2013: table 1).

## Supplementary Material

XML Treatment for Cucumis sativus L. var. xishuangbannanensis
